# Pharmacological Modulation of Cardiac Remodeling after Myocardial Infarction

**DOI:** 10.1155/2020/8815349

**Published:** 2020-12-30

**Authors:** Wei Zhao, Jia Zhao, Jianhui Rong

**Affiliations:** ^1^School of Chinese Medicine, Li Ka Shing Faculty of Medicine, University of Hong Kong, 10 Sassoon Road, Pokfulam, Hong Kong, China; ^2^Zhujiang Hospital, Southern Medical University, 253 Industrial Road, Guangzhou, Guangdong Province, China; ^3^Shenzhen Institute of Research and Innovation, The University of Hong Kong, Shenzhen, China

## Abstract

Cardiac remodeling describes a series of structural and functional changes in the heart after myocardial infarction (MI). Adverse post-MI cardiac remodeling directly jeopardizes the recovery of cardiac functions and the survival rate in MI patients. Several classes of drugs are proven to be useful to reduce the mortality of MI patients. However, it is an ongoing challenge to prevent the adverse effects of cardiac remodeling. The present review aims to identify the pharmacological therapies from the existing clinical drugs for the treatment of adverse post-MI cardiac remodeling. Post-MI cardiac remodeling is a complex process involving ischemia/reperfusion, inflammation, cell death, and deposition of extracellular matrix (ECM). Thus, the present review included two parts: (1) to examine the basic pathophysiology in the cardiovascular system and the molecular basis of cardiac remodeling and (2) to identify the pathological aspects of cardiac remodeling and the potential of the existing pharmacotherapies. Ultimately, the present review highlights drug repositioning as a strategy to discover effective therapies from the existing drugs against post-MI cardiac remodeling.

## 1. Introduction

Acute myocardial infarction (AMI), commonly referred to as a heart attack, is one of the most common cardiovascular diseases and a significant cause of morbidity and mortality, while 7.3 million deaths per year were estimated worldwide [1, 2]. The loss of functional myocardium characterizes the pathology of AMI. The myocardial injury initiates adaptive immune responses so that the heart undergoes structural and functional changes to maintain the cardiac outcome. Such changes in the heart are termed as cardiac remodeling [3]. Although cardiac remodeling was initially created to describe the anatomical changes in the left ventricle of the infarcted hearts, myocardial infarction (MI) is well-known to alter cardiac energy metabolism, impair intramyocardial perfusion, and attenuate diastolic and systolic functions [4].

Post-MI cardiac remodeling involves several pathophysiological processes, such as ischemia/reperfusion, cell death, inflammation, synthesis, and deposition of the extracellular matrix (ECM), resulting in changes in ventricular morphology, structure, and functions [5, 6]. It is an ongoing challenge to overcome the adverse effects of post-MI cardiac remodeling. Enormous effort has been made to develop effective targeted pharmacological therapies by targeting the basic pathophysiological processes [7–9]. Nevertheless, several existing treatments are often administered simultaneously to achieve satisfactory efficacy. Drugs, including adenosine, nicorandil, nitroprusside, and atrial natriuretic peptide and statins, are administered with other therapies for ischemia/reperfusion injury [10, 11]. Indeed, clinical experience in the past 30 years validated the effectiveness of several existing drugs and interventions to reduce the mortality of AMI patients. However, little is known about the pharmacological approaches for effectively controlling cardiac remodeling [5, 12, 13]. In clinical practice, drugs including angiotensin-converting enzyme inhibitors, angiotensin receptor blockers, aldosterone inhibitors, renin inhibitors, nicorandil, beta-blockers, and statins are administered chronically for effective treatment of the chronic phase of left ventricular remodeling [14, 15]. It appears that the combination therapy with two or more drugs (e.g., ACE inhibitors/AR blockers, aldosterone antagonist) is currently the preferred strategy for preventing post-MI adverse cardiac remodeling [6]. Therefore, the improvement of therapeutics for myocardial recovery should be achieved based on the comprehensive understanding and analysis of the pathobiology of cardiac remodeling [9, 16]. It is of great interest to identify novel molecular targets in the pathology of cardiac remodeling and dysfunction [17, 18].

The present review basically examined different key pathological aspects of cardiac remodeling and the existing pharmacotherapies in the past 10 years. The findings from this review may pave the avenue to develop effective therapeutics for treating the adverse effects of cardiac remodeling.

## 2. Key Pathological Alterations in Cardiac Remodeling

Cardiac remodeling is a complex process in which the pathologic stimuli alter cardiac structure, shape, and function [6]. Cardiac remodeling encompasses both the MI-triggered acute events within 90 min of an ST-elevation myocardial infarction as well as the long-standing events in the post-MI period of months or even years [6, 19–21]. In the early phase of MI, cardiac remodeling is driven by the infarct expansion [22]. Early changes are detectable within hours to days after the acute myocardial insult. Myocardial necrosis results in an influx of inflammatory cells, including macrophages and neutrophils [23]. The influx of different inflammatory cells leads to the destruction of the collagen scaffolding, leading to the alteration of ventricular shape, regional thinning, and dilation of the myocardium in the infarcted areas [24]. Over the following weeks to months, the viable myocardium is still challenged by different pathologic events including the activation of proteases and elevated expression of cytokines, especially those that may induce cardiomyocyte apoptosis and increase the release of the proinflammatory factor. The late phase involves reactive myocyte hypertrophy, interstitial fibrosis, and left ventricular dilatation [25]. Adverse cardiac remodeling is known to impair ventricular function and cause heart failure, representing a significant cause of mortality and morbidity in AMI patients [26]. However, the existing cardiovascular medicines are not designed to target cardiac remodeling due to the lack of understanding of the molecular mechanism of cardiac remodeling [4].

**Figure 1:**
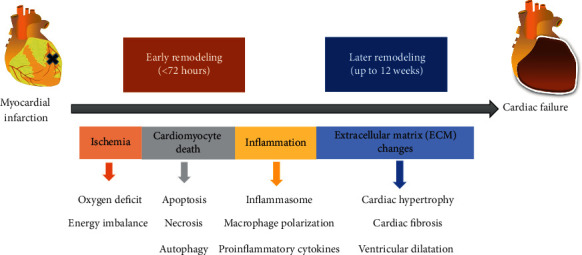
The phases and the main pathological changes in post-MI cardiac remodeling. The early remodeling includes ischemia, cell death, and inflammation within hours to days after the acute myocardial insult. The late remodeling is characterized by ECM deposition and causes reactive cardiac hypertrophy, cardiac fibrosis, and ventricular dilatation.

In fact, the post-MI remodeling process involves inflammatory, proliferative, and maturation phases. The pathologic responses in the infarcted heart include but not limited to ischemia, ischemia-reperfusion injury, inflammation (myocarditis), biomechanical stress, excess neurohormonal activation, excess afterload (hypertension, aortic stenosis), and cytokine storm [22, 26]. The post-MI phases and main pathological changes in cardiac remodeling are illustrated in Figure 1.

### 2.1. Ischemia in the Infarcted Heart

The basic pathology of MI is characterized by ischemia and cardiomyocyte death due to the imbalance between oxygen supply and demand [27, 28]. Myocardial ischemia markedly perturbs the ionic balance in cardiac metabolism [29]. Ischemia is known to cause the sudden cessation of oxidative phosphorylation and forces cardiomyocytes to overrun glycolysis for ATP production. Under physiological conditions, cardiomyocytes possess sufficient energy reserves to maintain contractility against short-time ischemia [30]. The prolonged ischemia disturbs myocardial metabolism and thereby reduces myocardial contractility. Indeed, myocardial ischemia not only causes systolic dysfunction but also reduces the compliance of the ventricle and diastolic dysfunction. When MI occurs, cardiomyocytes undergo necrosis due to initial ischemia and subsequent apoptosis during reperfusion as free radicals activate proapoptotic pathways [31, 32]. During adverse cardiac remodeling, the heart undergoes progressive ventricular dilatation, cardiac hypertrophy, fibrosis, and deterioration of cardiac performance. Thus, the outcome of cardiac remodeling is determined by interactions between the adaptive modifications and negative adaptations in cardiomyocytes [33].

### 2.2. Cardiomyocyte Death

The heart represents a well-organized assembly of cardiomyocytes, fibroblasts, endothelial, and smooth muscle cells. The death of cardiomyocytes directly causes post-MI heart failure [34, 35]. Upon MI, cardiomyocytes are damaged by necrosis, apoptosis, and autophagy. Several cellular pathways are activated to drive the progression from cardiac injury to adverse cardiac remodeling [4, 36]. Apoptosis, autophagy, and necroptosis are the regulated forms of cell death in infarcted hearts, although it is difficult to quantify the contributions of individual forms to the infarct size [37–39]. Progressive cell death directly causes cardiac remodeling in chronically overloaded hearts [6, 40]. Apoptosis is an energy-dependent form of cell death with DNA disintegration and without an associated inflammatory response. Apoptosis signal-regulating kinase (ASK) is the key mediator of cell death and susceptibility to heart failure in heart hypertrophy [5]. Necroptosis representing programmed necrosis is recently described as a novel form of regulated cell death and may play a prominent role in cardiovascular diseases [6]. With high relevance to necrosis and apoptosis, necroptosis is uniquely regulated by the activation of specific receptor-interacting protein kinases [41, 42]. Autophagy is another regulated form of cell death, characterized by the orderly degradation and recycling of cellular components, and upregulated in response to nutrient deprivation [43, 44], oxidative stress [45], and hypoxia [46]. A recent study showed that beclin-1 heterozygous deficiency protected mice from adverse cardiac remodeling in the model of I/R via regulating autophagy [47]. During the heart transplantation in mice, mechanical unloading of the LV activates autophagy and of FoxO3, leading to cardiac atrophy [48]. FoxO proteins are critical regulators of autophagy in the regression of cardiac hypertrophy after unloading the hypertrophic stimuli. Upregulation of FoxO proteins may be a novel therapeutic target to reverse cardiac hypertrophy via autophagy-mediated mechanism [33, 48]. Interestingly, the extent of activation determines whether autophagy protects cells from apoptotic death or promotes cell death [6, 49]. Presumably, apoptosis, necrosis, and autophagy coexist in many ways, such as in parallel and sequential in different sequences [50, 51]. Therefore, further investigation should define how much each type of cell death contributes to myocardial remodeling [4].

### 2.3. Inflammation

Myocardial ischemia and necrosis stimulate robust infiltration of leukocytes, which not only helps to clear necrotic debris but also triggers an intense organized inflammatory response [52, 53]. Such inflammatory cascade involves various components of the innate immunity and affects cardiomyocytes and noncardiomyocyte cells [31, 54]. During the first few days after MI, ischemic or nonischemic cardiomyocytes undergo necrosis and secret cytokines (e.g., tumor necrosis factor-*α* (TNF-*α*), interleukin-1 beta (IL-1*β*), and interleukin (IL-6). These cytokines recruit inflammatory cells to the site of injury, leading to adverse cardiac remodeling and dysfunction [4, 53, 55]. Excessive inflammatory response induces myocardial apoptosis and promotes cardiac pathological remodeling [56]. On the other hand, some of the immune cells may contribute to the wound-healing process for post-MI cardiac repair [56].

**Figure 2:**
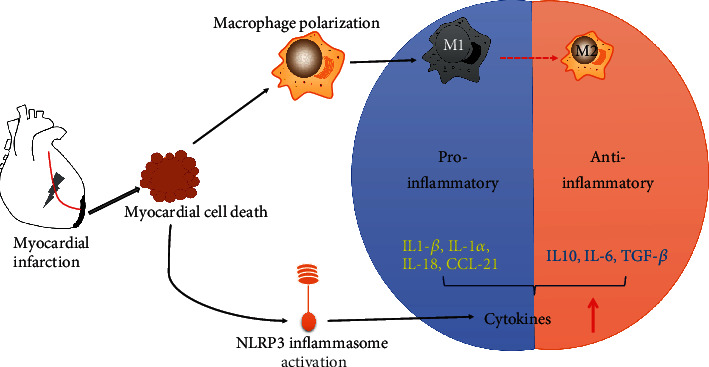
Macrophage M1/M2 polarization and the inflammatory response in MI. MI-induced inflammatory response includes the initial proinflammatory and the subsequent anti-inflammatory reparative phase. In the proinflammatory phase, macrophages undergo M1 polarization while activation of the NLRP3 inflammasome increases the release of proinflammatory cytokines (such as IL-1*β*, IL-18, IL-1*α*, CCL21). In the subsequent anti-inflammatory reparative phase, macrophages undergo M2 polarization while anti-inflammatory factors (such as IL-10, IL6, TGF-*β*) are produced to execute the resolution of cardiac inflammation.

Post-MI inflammation plays a critical role in determining AMI size and post-MI adverse cardiac remodeling. The detrimental effects of AMI are mediated by several inflammatory mediators, which are important therapeutic targets for cardiac protection [57, 58]. Firstly, the NACHT, LRR, and PYD domain-containing protein 3 (NLRP3) inflammasome is an important ubiquitous intracellular pattern recognition receptor for regulating the inflammatory response during AMI [59]. Particularly, the NLRP3 inflammasome determines the production of IL-1*β* and ensures systemic inflammatory response. In the early phase of inflammation, the inflammasome specks are detectable in the endothelial cells, cardiomyocytes, and fibroblasts. The expression of NLRP3 and the activity of the inflammasome in the heart were low within 3 h after AMI in a mouse model of ischemia-reperfusion injury [60, 61]. In this model, the NLRP3 inflammasome was formed in the myocardium within 3–24 h after AMI, contributed to the inflammatory response, and exacerbated the ischemia-reperfusion injury [60]. Importantly, genetic and pharmacological inhibitions of the inflammasome-related components (e.g., caspase 1, IL-1*β*, ASC, and NLRP3) are effective to reduce MI size [62–66]. The MI-induced inflammatory response is schematically illustrated in Figure 2.

Neutrophils are well-known to be the first-line defender against invading microorganisms. Within the context of MI, neutrophils are activated to generate oxygen free radicals and secrete chemokines and matrix metalloproteinases (MMPs) [67]. Dysregulation of neutrophils may affect cardiac remolding due to overproduction of free radicals, insufficient phagocytosis of cell debris, and aberrant degradation of the extracellular matrix.

Secondly, macrophages are known to play a significant role in the pathophysiology of MI, while the timely switch of macrophage polarization is a potential therapeutic target for promoting myocardial healing [62, 68, 69]. Macrophage phenotypes are classified into activated macrophages (M1) and alternatively activated macrophages (M2) [70]. Depending on the pathological environment, M2 macrophages are further differentiated into the following four polarization subtypes: M2a, M2b, M2c, and M2d. Gleissner et al. described another M4 macrophage, which is different from phenotypes M1 and M2 [71]. Although several factors have been identified for regulating macrophage polarization, little is known about the spatiotemporal relationships and functions of the various macrophage subsets in the post-MI cardiac remodeling [72, 73]. A recent study demonstrated that cardio sphere-derived cells decreased proinflammatory M1 macrophages but increased anti-inflammatory M2 macrophages in the infarcted hearts, thereby supporting heart repair [74]. *In vivo* IL-10 infusion significantly improved post-MI cardiac physiology and increased cardiac macrophage M2 polarization and fibroblast activation to moderate collagen deposition [74]. Therefore, pharmacological modulation of monocyte differentiation and macrophage polarization could provide anti-inflammatory and reparative therapeutic strategies for the treatment of post-MI cardiac remodeling [62].

### 2.4. Changes in Cardiac ECM

Cardiac ECM serves as the cellular scaffold to maintain the shape and geometry of the heart [75]. Biochemically, ECM represents a complex network of different cellular components including collagen, MMPs, and cell surface adhesion molecules [76]. The composition of the ECM components determines cardiac remodeling in the infarct heart. In the early phase after MI, cardiac fibroblasts migrate to the infarcted area in response to cytokines, synthesize and secrete ECM components (mainly collagen) to replace the necrotic tissues, stabilize the cardiac structure, and prevent cardiac rupture. Uncontrolled deposition of ECM components promotes the formation of myocardial fibrosis, decreases ventricular compliance, and causes cardiac dysfunction and heart failure over a long time. Moreover, cardiac fibrosis further stimulates the electrophysiological remodeling, inducing arrhythmogenesis and affecting the quality of life.

Different endogenous factors dynamically regulate the synthesis and degradation of ECM components. The healthy cardiac ECM is primarily composed of type I collagen (70%) and type III collagen (12%). Type I, III, IV, V, and VI collagen are increased in the infarct region, while type I collagen and type III collagen are the major components of the myocardial scar [77, 78]. Moreover, ischemia and reperfusion are known to profoundly increase several other ECM proteins, including intermediate cartilage layer protein 1 (CILP1), asporin, adipocyte enhancer-binding protein 1 (AEBP1), and transforming growth factor *β*-induced gene-h3 (TGFBI) in the border zone at 15 days after reperfusion in a porcine model of ischemia/reperfusion injury [79]. Collectively, the remodeling of the myocardium structure involves the excessive accumulation of fibrillar collagen matrix in the form of “reparative” and “reactive” fibrosis, causes adverse consequences on ventricular function and arrhythmogenicity, and supports the pathophysiological concept of interstitial heart disease [56].

The proliferative phase of cardiac remodeling is hallmarked by scar formation at 2–7 days for mice and 4–14 days for humans, respectively. Pharmacological intervention is needed to limit pathological ECM remodeling and promote infarct repair. Collagen-based biomaterials may provide mechanical support, improve angiogenesis and tissue integration, reduce inflammation and apoptosis, and limit adverse remodeling and the loss of cardiac function in the animal MI models [80, 81]. On the other hand, recombinant human collagen was also evaluated for the preparation of clinically relevant biomaterials to reduce pathological remodeling and ameliorate post-MI cardiac functions [82].

## 3. Pharmacological Approaches

Cardiac remodeling contributes to the development and progression of ventricular dysfunction, arrhythmias, and poor prognosis. Uncontrolled cardiac remodeling is a significant cause of mortality and morbidity in AMI patients [67]. Therefore, adverse cardiac remodeling is an important therapeutic target for the treatment of AMI. In fact, the basic pathophysiological processes are targeted for the development of pharmacological treatments [83, 84]. Different existing drugs including *β*-adrenergic receptor (*β*-AR) blockers, angiotensin-converting enzyme (ACE) inhibitors, angiotensin-receptor (AR) blockers, mineralocorticoid receptor blockers, hydralazine, nitrates, and cardiac resynchronization therapy have been evaluated for preventing adverse cardiac remodeling [22, 85].

### 3.1. *β*-AR Blockers

The activation of *β*-ARs increases the cAMP synthesis and activates protein kinase A, representing the primary mechanism for acute enhancement of cardiac reserve to maintain heart function [6]. The *β*-AR blockers suppress the activation of *β*-ARs and attenuate adverse cardiac remodeling at the molecular and organ levels [86]. Indeed, several *β*-AR blockers exhibit potential for reversing cardiac remodeling, although such results should be further validated by clinical trials [87]. A recent report suggested that *β*-AR blockers reduced the profibrotic potential of resident cardiac progenitor cells in patients [88]. Interestingly, excessive adrenergic stimulation may also affect the *in situ* myofibroblastic potentials of resident progenitors through *β*2-AR signaling, resulting in detrimental profibrotic outcomes [89]. Acute overactivation of *β*-ARs induces inflammasome-dependent production of IL-18 within the myocardium while activates cytokine cascades, macrophage infiltration, and pathological cardiac remodeling [90]. Neutralizing IL-18 at the early stage of *β*-AR activation successfully prevented inflammatory responses and cardiac injuries. *β*-adrenergic stimulation on the *β*-AR-G*α*s/Src pathway activates the STAT3 pathway for maintaining normal cardiac function and minimizing adverse cardiac remodeling [91]. Cardiac-specific overexpression of *β*3-AR inhibits the hypertrophic response to neurohormonal stimulation through a NOS-mediated mechanism [92]. Thus, *β*3-AR agonists may have therapeutic potential for the modulation of cardiac remodeling.

### 3.2. ACE Inhibitors and AR Blockers

**Figure 3:**
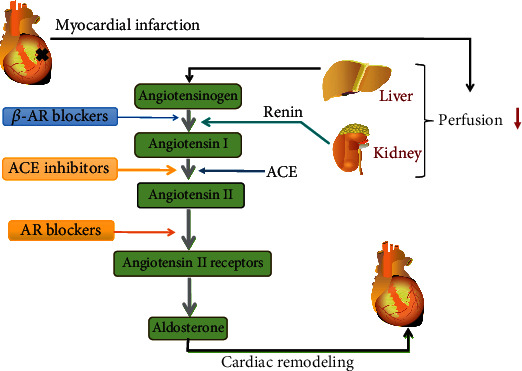
MI-induced activation and pharmacologic inhibition of the renin-angiotensin-aldosterone system. Renin-angiotensin-aldosterone system regulates the functions of multiorgans. AMI causes a decrease in cardiac output, reduces tissue perfusion in the kidney and liver, and stimulates renin and angiotensinogen production. Consequently, angiotensin production is increased to stimulate ventricular remodeling. Meanwhile, aldosterone has similar effects on cardiac remodeling.

The renin-angiotensin system regulates the structural remodeling of the left ventricle for post-MI cardiac healing and long-term prognosis [92]. Interestingly, the renin-angiotensin system is regulated by multiple mechanisms: (1) *β*-AR blockers inhibit the secretion of renin, (2) renin inhibitors directly diminish the activity of renin, (3) ACE inhibitors block the formation of angiotensin II, and (4) AR blockers dampen the activation of angiotensin II type 1 (AT1) receptor. As shown in Figure 3, these pharmacological mechanisms may inhibit the renin-angiotensin system in a synergistical manner.

It is well-known that acute activation of the renin-angiotensin-aldosterone system alleviates hemodynamic stress. Dysregulation of the renin-angiotensin-aldosterone system is often associated with hypertension, oxidative stress, and adverse cardiac remodeling [93]. The sustained release of angiotensin promotes cardiac fibrosis, cellular necrosis, and cardiomyocyte hypertrophy. Indeed, agents that inhibit either the renin-angiotensin-aldosterone system or sympathetic nervous system reduced mortality in MI patients [94]. ACE inhibitors are widely used in the management of heart failure in the last three decades [95]. Several randomized clinical trials demonstrated the beneficial effect of ACE inhibitors, mineralocorticoid receptor blockers, and AR blockers in the management of heart failure patients [25]. These drugs act at the different points in the signaling cascade of angiotensin II to strop adverse cardiac remodeling regardless of the changes in blood pressure [96]. Others showed that ACE inhibitors favourably altered the loading conditions for LV, reduced progressive LV remodeling, and improved clinical outcomes [97, 98]. AR blockers block the effects of angiotensin II particularly at the receptor subtype 1 level to mediate vasoconstriction, sodium and water retention, cardiac hypertrophy, and cardiac fibrosis [99]. It is noteworthy that randomized trials have not approved the superiority of AR blockers over ACE inhibitors, although angiotensin II antagonism is theoretically favourable in patients with AMI [10, 100].

On the other hand, the stimulation of the renin-angiotensin-aldosterone system leads to the activation of different MMPs in the heart and subsequent degradation of extracellular proteins in the myocardium [37, 101]. MMP-2 and MMP-9 are two typical MMPs for ECM degradation and post-MI cardiac remodeling [102, 103]. Pharmacological inhibition of MMPs is sufficient to limit tissue damage in animal MI models [104, 105].

### 3.3. Anti-Inflammatory Agents

Inflammation is a critical driving force for post-MI ventricular remodeling [106]. Unrestrained inflammation induces matrix degradation and cardiomyocyte apoptosis in the infarcted myocardium [107]. Prolonged inflammation is known to promote LV dilation, impair LV physiology, and enhance excessive scar formation [108]. Thus, modulation of the inflammatory response represents a potential strategy for intervening post-MI cardiac remodeling. Timely and effective suppression of inflammatory signaling in infarcted hearts is of great importance to protect the myocardium from dilative remodeling and progressive cardiac dysfunction [109]. Indeed, inhibition of the initial proinflammatory responses and promotion of the subsequent anti-inflammatory reparative responses are essential therapeutic strategies to limit MI size and prevent adverse LV remodeling [62]. Therefore, timely resolution of cardiac inflammation is critical to control cardiac remodeling [53].

Therapeutic targets are primarily several cytokines that control the initial proinflammatory response and promote the subsequent anti-inflammatory reparative response. Firstly, IL-1 is an important proinflammatory cytokine for the treatment of atherosclerosis, AMI, and heart failure [110]. Interestingly, blockade of IL-1*β* signaling limits adverse cardiac remodeling after AMI [111, 112]. IL-1*α* is released from the damaged cardiomyocytes and activates IL-1R1 on cardiac fibroblasts, inducing early myocardial remodeling in AMI [111]. Preclinical research and clinical trials validated the role of IL-1*α* in the initiation of post-MI inflammation and the role of IL-1*β* in adverse cardiac remodeling and heart failure [63]. Secondly, the CC-chemokine CCL21 interacts with receptor CCR7 to regulate inflammation and immune cell recruitment in response to pressure overload in symptomatic aortic stenosis [113]. Thirdly, IL-10 is an anti-inflammatory cytokine for resolving cardiac inflammation [114]. Myocardial ischemia and reperfusion significantly increased IL-10 in the serum within 6 h in animal models [53]. Finally, the NLRP3 inflammasome is a multiprotein complex for regulating the proteolytic activation of caspase 1 and proinflammatory cytokines such as IL-1*β* and IL-18 [64]. NLRP3 inhibition significantly reduced infarct size and preserved cardiac function, suggesting that the early activation of NLRP3 inflammasome may control the downstream inflammatory signaling [115].

### 3.4. Miscellaneous Aspects

#### 3.4.1. Circadian Rhythm Regulation

Circadian rhythm is known to regulate various biological and cardiovascular rhythms in health and disease and modulates post-MI cardiac remodeling and dysfunction [116, 117]. Circadian rhythms control myocardial homeostasis, apoptosis, autophagy, and necrosis [51]. Circadian rhythm is closely associated with the systems that modulate oxidative stress and may thereby modulate post-MI cardiac remodeling [118]. Melatonin is a well-known circadian rhythm regulator and antioxidant. Several studies demonstrated the cardioprotective activity of melatonin in AMI [119, 120].

#### 3.4.2. Noncoding RNAs (ncRNAs)

ncRNAs include small ncRNAs and long ncRNAs (lncRNA) while miRNAs are a class of small ncRNAs [121]. Intravenous miR-144 administration decreased the left ventricular remodeling in post-MI hearts. Further analysis revealed that miR-144 decreased myocardial fibrosis, inflammation, and apoptosis by regulating the autophagy signaling pathways [122]. The lncRNA Wisper reduced the cardiac fibrosis and prevented myocardial remodeling in post-MI hearts [123]. Downregulation of lncRNA Neat1 and AK139328 alleviated the myocardial ischemia/reperfusion injury via regulating the autophagy signaling [124]. The lncRNA Mhrt also protected the heart from cardiac hypertrophy by ATP-dependent chromatin-remodeling [125].

#### 3.4.3. Gut Microbiota

Dysbiosis of the gut microbiota has recently emerged as a novel target for the control of cardiac remodeling [126]. Gut microbiota composition is linked to cardiovascular diseases (CVD) through multiple mechanisms including (1) direct effects of microbial metabolites on atherosclerosis and thrombosis and (2) immune modulation by bacteria and their products [127]. Probiotics may change gut microbiota composition to achieve the maximal beneficial effect on the cardiac remodeling process in MI patients. Lam et al. confirmed the potential effects of microbiota on the post-MI ventricular remodeling [128]. Oral administration of antibiotic vancomycin and probiotic Good belly was containing Lactobacillus plantarum and Bifidobacterium lactis Bi-07 before ischemia/reperfusion (I/R) injury significantly reduced infarct size and improved myocardial function in rats [129]. An increasing number of recent studies validated the link of gut microbiota to myocardial function and repair in AMI [130]. Selective gut modulation by probiotic administration improved metabolic dysfunction and attenuated cardiac remodeling in AMI [131].

#### 3.4.4. Antibiotics

Antibiotics may affect ventricular remodeling in the immediate post-MI setting. The “PROVE IT-TIMI22 trial” demonstrated that long-term treatment with gatifloxacin could prevent major adverse cardiovascular events in the subjects with recent acute coronary syndrome [132]. Doxycycline attenuated adverse ventricular remodeling via inhibiting MMPs [133]. Experimental and clinical studies suggested that cyclosporine could attenuate reperfusion injury and prevent adverse left ventricular remodeling [134]. Mechanistically, cyclosporine is a pharmacologic inhibitor of cyclophilin D, a central regulator for the opening of the mitochondrial permeability transition pore (mPTP). Indeed, either genetic or pharmacologic inhibition of cyclophilin D reduced the severity of myocardial reperfusion injury [135, 136].

## 4. Conclusion and Perspectives

The present review is aimed at identifying drugs for effective treatment of adverse cardiac remodeling in MI. We have summarized the pharmacological therapies on cardiac remodeling after AMI (Table 1). Firstly, the complex pathophysiological processes of cardiac remodeling were investigated to understand better the interactions between the cellular components, signaling molecules, the ECM components, and neurohormonal regulation. Secondly, the capacity of several classes of the existing drugs was assessed to target the vital pathological processes in cardiac remodeling. Surprisingly, different drugs should be combined to achieve efficacy in selected patients. Therefore, a better understanding of the complex pathological process of cardiac remodeling provides the key to develop more effective and safe strategies for the treatment of post-MI cardiac remodeling.

## Figures and Tables

**Table 1:** Summary of pharmacological therapies against adverse post-MI cardiac remodeling.

Drug/target	Mechanism of action	Drug application phase	References
*β*-AR blockers	Prevent *β*-ARs/desensitization	Clinical	[86, 87, 89, 90]
ACE inhibitors	Inhibit angiotensin-converting enzyme	Clinical	[95, 97, 98]
AR blockers	Inhibit angiotensin receptor/desensitization	Clinical	[25, 99, 100]
Anti-inflammatory agents	Resistance to an excessive inflammatory response	Clinical	[106, 107, 110, 115]
Probiotics	Regulate gut microbiota	Preclinical	[126, 127, 130, 131]
Antibiotics	Inhibit matrix metalloproteinase/opening mPTP	Clinical	[132–134, 136]
Circadian rhythm regulators	Control cell fate/modulate oxidant stress	Preclinical	[117, 118, 120]
Noncoding RNAs (ncRNAs)	Reduced the cardiac fibrosis/regulating the autophagy signaling	Preclinical	[121–123, 125]
